# Insight into the acid tolerance mechanism of *Acetilactobacillus jinshanensis* subsp. *aerogenes* Z-1

**DOI:** 10.3389/fmicb.2023.1226031

**Published:** 2023-07-14

**Authors:** Qin Li, Kaidi Hu, Juan Mou, Jianlong Li, Aiping Liu, Xiaolin Ao, Yong Yang, Li He, Shujuan Chen, Likou Zou, Mingye Guo, Shuliang Liu

**Affiliations:** ^1^College of Food Science, Sichuan Agricultural University, Ya’an, Sichuan, China; ^2^College of Resources, Sichuan Agricultural University, Chengdu, Sichuan, China; ^3^Sichuan Baoning Vinegar Co., Ltd, Langzhong, Sichuan, China

**Keywords:** vinegar brewing, *Acetilactobacillus jinshanensis* subsp. *aerogenes* Z-1, acidic stress, acid resistance, acid tolerance-related gene

## Abstract

Several lactic acid bacteria (LAB) are double-edged swords in the production of Sichuan bran vinegar; on the one hand, they are important for the flavour of the vinegar, but on the other hand, they result in vinegar deterioration because of their gas-producing features and their acid resistance. These characteristics intensify the difficulty in managing the safe production of vinegar using strains such as *Acetilactobacillus jinshanensis* subsp. *aerogenes* Z-1. Therefore, it is necessary to characterize the mechanisms underlying their acid tolerance. The results of this study showed a survival rate of 77.2% for Z-1 when exposed to pH 3.0 stress for 1 h. This strain could survive for approximately 15 days in a vinegar solution with 4% or 6% total acid content, and its growth was effectively enhanced by the addition of 10 mM of arginine (Arg). Under acidic stress, the relative content of the unsaturated fatty acid C18:1 (n-11) increased, and eight amino acids accumulated in the cells. Meanwhile, based on a transcriptome analysis, the genes *glnA*, *carA/B*, *arcA*, *murE/F/G*, *fabD/H/G*, *DnaK*, *uvrA*, *opuA/C*, *fliy*, *ecfA2*, *dnaA* and *LuxS*, mainly enriched in amino acid transport and metabolism, protein folding, DNA repair, and cell wall/membrane metabolism processes, were hypothesized to be acid resistance-related genes in Z-1. This work paves the way for further clarifying the acid tolerance mechanism of Z-1 and shares applicable perspectives for vinegar brewing.

## 1. Introduction

Brewing vinegar, as a liquid condiment, is made from various materials containing starch or sugar through microbial fermentation. There are more than 40 genera of microorganisms involved in vinegar fermentation, including moulds, yeasts, acetic acid bacteria, *Bacillus*, and lactic acid bacteria (LAB) (Nie et al., 2015). Among them, LAB participate throughout the entire brewing process and are dominant in the bacterial community. Various species of lactic acid bacteria occur in different fermentation stages. For example, *Lactococcus* and *Weisellosis* mainly appear in the koji-making stage, while *Lactobacillus* is dominant in vinegar culture (Li et al., 2016; Gan et al., 2017).

Sichuan bran vinegar is one of the four well-known solid fermented vinegars in China. Compared with the other three, a higher content of lactic acid than acetic acid is one major feature of Sichuan bran vinegar (Al-Dalali et al., 2020). The lactic acid generated by LAB neutralizes the pungent acidity of acetic acid and awards the vinegar a soft taste, and subsequent esterification can further enrich the flavour. However, owing to the open-scale environment, solid fermentation is also vulnerable to contamination by microorganisms. The swelling of vinegar triggered by LAB through gas production has also been reported (Zhang et al., 2016; Wang et al., 2023). A case of swelling deterioration of Sichuan bran vinegar occurred in 2016, causing serious adverse effects, such as product stability, long detection time and increased production costs. Moreover, these gas-producing LAB also demonstrate acid resistance and are present throughout the entire vinegar fermentation process, which makes it more difficult to produce vinegar safely. In our previous work, a gas-producing LAB named Z-1 was isolated from spoiled Sichuan bran vinegar and identified as *Acetilactobacillus jinshanensis* subsp. *aerogenes* Z-1 demonstrated acid tolerance, and the optimal pH for its growth was pH 3.6 (Wang et al., 2023). Although it has been shown that the source of swelling deterioration in Sichuan bran vinegar is the presence of this Lactobacillus species throughout the vinegar fermentation process, together with its acid resistance, the structure of a comprehensive strategy to address it remains unclear. Therefore, it is necessary to characterize the mechanism underlying the acid resistance of Z-1. In the present work, the acid tolerance characteristics of strain Z-1 were explored from different aspects, including acid tolerance ability, growth characteristics in vinegar solution, the effect of exogenous amino acids, and physiological responses. Furthermore, transcriptome analysis was employed to identify the genes involved. It is hoped that this study will shed light on the acid tolerance mechanism of strain Z-1.

## 2. Materials and methods

### 2.1. Microorganisms and media

*Acetilactobacillus jinshanensis* subsp. *aerogenes* strain Z-1 was preserved at −80°C by the Food Microbiology Laboratory of Sichuan Agricultural University and deposited in the General Microbiology Center of the China Microbial Culture Collection Management Committee (No. CGMCC 20399).

The inoculum of strain Z-1 was prepared by incubation at 33°C for 3 days in modified MRS medium (20 g/L glucose, 10 g/L peptone, 10 g/L beef extract, 5 g/L yeast extract powder, 2 g/L dipotassium phosphate, 5 g/L sodium acetate, 0.2 g/L magnesium sulfate, 0.05 g/L manganese sulfate, 2 g/L Tween-80, 2 g/L dibasic ammonium citrate, 0.5–5 g/L compound amino acid, 0.3–3 g/L compound vitamin B, 0.2–1 g/L nucleotides, and 1–5 g/L compound growth factors; pH adjusted to 3.6 with lactic acid:acetic acid = 1:1) (Wang et al., 2023). The pH of all modified MRS media were adjusted with lactic acid:acetic acid = 1:1.

6° vinegar: 6% total acid content (6.00 g/100 mL vinegar, pH 3.5) was provided by a vinegar manufacturer located in Sichuan, southwest China; 1% glucose was added prior to use, and the mixture was subjected to autoclaving in a boiling water bath for 30 min.

4° vinegar: 4% total acid content (a mixture of 6° vinegar and water, v/v 2:1, pH 3.5); 1% glucose was added prior to use and subjected to autoclaving in a boiling water bath for 30 min.

### 2.2. Acid tolerance of strain Z-1

After transferring 1% (v/v) inoculum into 100 mL fresh modified MRS, the culture was incubated at 33°C for 65 h, achieving an OD_600_ of approximately 3.5. Then, 10 mL of culture was centrifuged (8,000 g/min, 10 min) at 4°C. The obtained biomass was washed twice using sterile saline and subjected to further incubation in 10 mL of modified MRS medium with the pH adjusted to 2.5, 2.8, 3.0, 3.2, and 3.6. Samples were withdrawn at designated times and subjected to serial dilution with sterile saline for viable cell counting. The dilute solution was spread on a modified MRS Petri dish, followed by 7–10 d of anaerobic incubation at 33°C. Each test was performed in triplicate.

### 2.3. Growth characteristics of Z-1 under acidic stress

Ten millilitres of inoculum was centrifuged (8,000 g/min, 10 min), and the resultant pellets were resuspended in plastic vials containing 100 mL of 4° and 6° vinegar. Then, the culture was incubated at 33°C. Samples were taken periodically for viable cell counting as described above. Triplicate experiments were carried out in parallel.

### 2.4. Effect of amino acids on the growth of Z-1

Batch experiments were performed in 250 mL Erlenmeyer flasks containing 100 mL modified MRS medium, where aspartic acid (Asp), glutamic acid (Glu), arginine (Arg) and lysine (Lys) were fortified individually at 10 mM, 20 mM, 30 mM and 40 mM. After inoculation (2%, v/v), the cultures were incubated at 33°C for 108 h. The substrate concentration was selected as an independent variable. Nonspiked medium was used as a control. All treatments were conducted in triplicate. Samples were withdrawn at 12-h intervals to measure the absorbance at 600 nm.

Then, the optimal concentration of each amino acid was selected for another batch following the same protocol. However, the medium was adjusted to different pH values (3.6, 3.4 and 3.2). The cell density of each culture was quantified at an interval of 12 h using the OD600.

### 2.5. Physiological response of Z-1 to acidic stress

After transferring 2% (v/v) inoculum into 250 mL Erlenmeyer flasks containing 100 mL fresh modified MRS (pH 3.6), the culture was incubated at 33°C for 65 h, achieving an OD_600_ of approximately 3.5, and then 50 mL of culture was centrifuged (8,000 g/min, 10 min) at 4°C. The harvested cells were washed twice using sterile saline and subsequently resuspended in 50 mL modified MRS medium at pH 3.0 and 3.6 (control) and maintained for 1 h at 33°C. All tests were performed in triplicate. Afterwards, samples were taken for analyses, including scanning electron microscopy (SEM), cellular membrane fatty acid content, H^+^-ATPase activity, intracellular ATP content, intracellular pH (pH_i_), and intracellular amino acid content.

Field emission scanning electron microscopy (FESEM) images were collected on an Evo 18 scanning electron microscope. The samples were prepared following the method of Zhang et al. (2010).

The fatty acid content of the cell membrane was determined through gas chromatography–mass spectrometry (GC–MS), and sample pretreatment and measurement were carried out according to Wu et al. (2012b).

H^+^-ATPase activity was determined using a Cell ATP bioluminescence quantitative detection kit (GMS10050, Genmed Scientifics Inc. United States), and ATP content was determined using a Bacterial H^+^-ATPase activity colorimetric assay kit (GMS50244.3, Genmed Scientifics Inc. United States). One U (μmol/min) of H^+^-ATPase activity is defined as the amount of enzyme that catalyses the conversion of 1 μM of reduced NADH per minute at 37°C. ATP content was defined as nmol/mg intracellular protein. The protein concentration was determined using a protein quantification test kit (Sangon Biotech, China).

pH_i_ was measured by the fluorescence method using 5- (and 6-)-carboxyfluorescein succimidyl ester as the fluorescent probe, that loading of cells with 5- (and 6-)-carboxyfluorescein succimidyl ester, determination of pH_i_, and calibration of pH_i_ all followed Breeuwer et al. (1996). Calibration curves establishing the relationship between extracellular pH and intracellular pH were established to exclude artifacts caused by environmental conditions (Breeuwer et al., 1996).

After acid stress treatment, 30 mL of treated culture was removed and centrifuged (4°C, 8000 g/min) for 10 min to collect the bacteria. The pellets were resuspended in 1 mL of 50 mM PBS buffer (pH 7.0) after washing 3 times with the same buffer. Then, the samples were boiled in water for 15 min and centrifuged (4°C, 8000 g/min) for 10 min to obtain the supernatant for protein content quantification. An equal volume of 20% (m/v) sulfosalicylic acid was added to 0.5 mL of supernatant. The mixture was maintained at 4°C for 1 h and then centrifuged (4°C, 8000 g/min) for 5 min, followed by filtration with a 0.22 μm water phase filter membrane. An automatic Hitachi L-8900 amino acid analyser was employed for analysis.

### 2.6. RNA sequencing and transcriptomics analysis

To perform RNA sequencing and transcriptomics analysis, strain Z-1 was first exposed to acid stress. After transferring 2% (v/v) inoculum into fresh modified MRS (pH 3.6), the culture was incubated at 33°C for approximately 65 h, achieving an OD_600_ of approximately 3.5, and then 50 mL of culture was centrifuged (8,000 g/min, 10 min) at 4°C. The harvested cells were washed twice using sterile saline and subsequently resuspended in 50 mL modified MRS medium at pH 3.0 and 3.6 (control) and maintained for 1 h at 33°C. The biomass was harvested through centrifugation (8,000 g/min, 10 min) at 4°C and washed twice using sterile saline. Then, the biomass was flash frozen using liquid nitrogen for RNA extraction. Each library was constructed in triplicate.

RNA extraction, transcriptomics sequencing and bioinformatics analysis were performed by Shanghai Majorbio Biopharm Technology Co., Ltd. (China). Total RNA was extracted using TRIzol Reagent (Invitrogen, United States), and the total RNA quality was determined using a Nanodrop spectrophotometer (Thermo Fisher Scientific, United States). The validated RNA underwent purification, fragmentation, reverse transcription, end repair, amplification, and circularization successively to obtain a library according to the company’s standard guidelines. A bioanalyzer was employed for quality control. Finally, whole-run sequencing was performed on Roche 454 GS FLX Titanium instrument (Roche Diagnostics, Indianapolis, IN, USA). High-quality reads of the transcriptome in each sample were aligned to the whole genome of strain Z-1 using the Bowtie 2 program. The gene expression level was quantified by RSEM software in transcripts per kilobase per million mapped reads (TPM) values. Next, differential expression analysis between two conditions/groups was carried out using edgeR (v2.12), DESeq2 (v3.11) and DESeq (v3.11) software. Fold change (FC) ≥ 1.8 and adjusted *p* value ≤0.05 were considered criteria for screening significant differentially expressed genes (DEGs) between different groups. All DEGs were searched against the nonredundant protein data in the NCBI, GO, Swiss-Prot and EggNOG databases, accompanied by cSNP/InDel screening.

### 2.7. RT–qPCR verification

To confirm the reliability of the RNA-Seq analysis, RT–qPCR was performed for 10 DEGs with the primers listed in Supplementary Table S1. Total RNA was extracted using a commercial kit (Sangon Biotech, China) coupled with DNase I (TaKaRa). RNA integrity was assessed using a 1.0% agarose gel. First-strand cDNA was synthesized using a RevertAidTM kit (Thermo Fisher Scientific, United States) following the manufacturer’s instructions. 16S rRNA served as an internal control. Each reaction was performed in a 20 μL system containing 10 μL of 2 × SG Fast qPCR Master Mix, 2 μL of cDNA sample, 0.4 μL of each primer, 2 μL of DNF Buffer and 5.2 μL of sterile ddH_2_O. The PCR protocol included a step of 95°C for 2 min, followed by 40 cycles of 95°C for 5 s, 60°C for 20 s, and 95°C for 15 s. Three biological replicates were performed for each reaction.

## 3. Results and discussion

### 3.1. Acid tolerance of strain Z-1

To assess the ability of strain Z-1 to tolerate acid, acid stress was imposed at different levels. The results are shown in Table 1. In comparison with the control group (pH 3.6), the number of viable cells was reduced to 8.09 lg CFU/mL at pH 3.2 at 0.5 h, and it further declined to 7.24 lg CFU/mL if the treatment was extended to 1 h. At pH 3.0, the number of viable cells at 0.5 h and 1 h were 7.49 lg CFU/mL and 6.75 lg CFU/mL, respectively. It is suggested that Z-1 was able to adapt to pH 3.0 ~ 3.6 with survival rates of more than 75% and microbial growth was completely eliminated at pH values below 3.0, which is consistent with a previous report (Wang et al., 2023). It shares a similar pH growth range with a previously reported strain, *Lactobacillus jinshani* sp. nov. HSLZ-75 (Yu et al., 2020).

**Table 1:** Viable cell counting of Z-1 at different pH.

Time (h)	pH	Viable cell counting (lg CFU/mL)
0.5	3.6	8.15 ± 0.048
	3.2	8.09 ± 0.045
	3.0	7.49 ± 0.049
	2.8	0
	2.5	0
1	3.6	8.74 ± 0.135
	3.2	7.24 ± 0.196
	3.0	6.75 ± 0.09
	2.8	0
	2.5	0
0(Control)	3.6	8.65 ± 0.029

### 3.2. Growth characteristics of Z-1 under acidic stress

**Figure 1:**
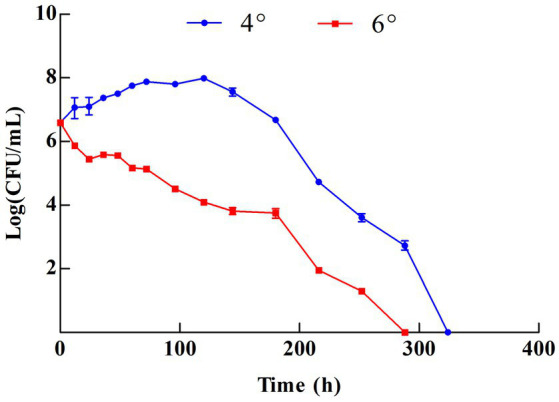
Growth of Z-1 in different vinegar solutions.

The growth status was observed at 4° and 6° based on the major grades of vinegar sold on the market (Figure 1). Z-1 kept growing in the 4° vinegar solution and reached a maximum biomass of 9.6 × 10^7^ CFU/mL at 120 h. Afterwards, the biomass started to decrease. In contrast, Z-1 could not grow in the 6° vinegar solution, demonstrating a decline in biomass during incubation. There were no viable cells after approximately 15 days in either culture. Z-1 may subsequently enter viable but non-culturable (VBNC) status, it is believed to be a survival strategy of bacteria to avoid adverse effects. This state can be revived under appropriate conditions, for example, strain Z-1 cannot grow with ordinary MRS medium during the early screening and can be isolated after adjusting the composition of the MRS medium. To date, several LAB have been reported to be able to survive under acidic stress and to harbour acid tolerance ability, which is mainly gained through external substances, cell membrane structure and intracellular metabolism (Wang et al., 2018).

### 3.3. Effect of amino acids on the growth of Z-1

**Figure 2:**
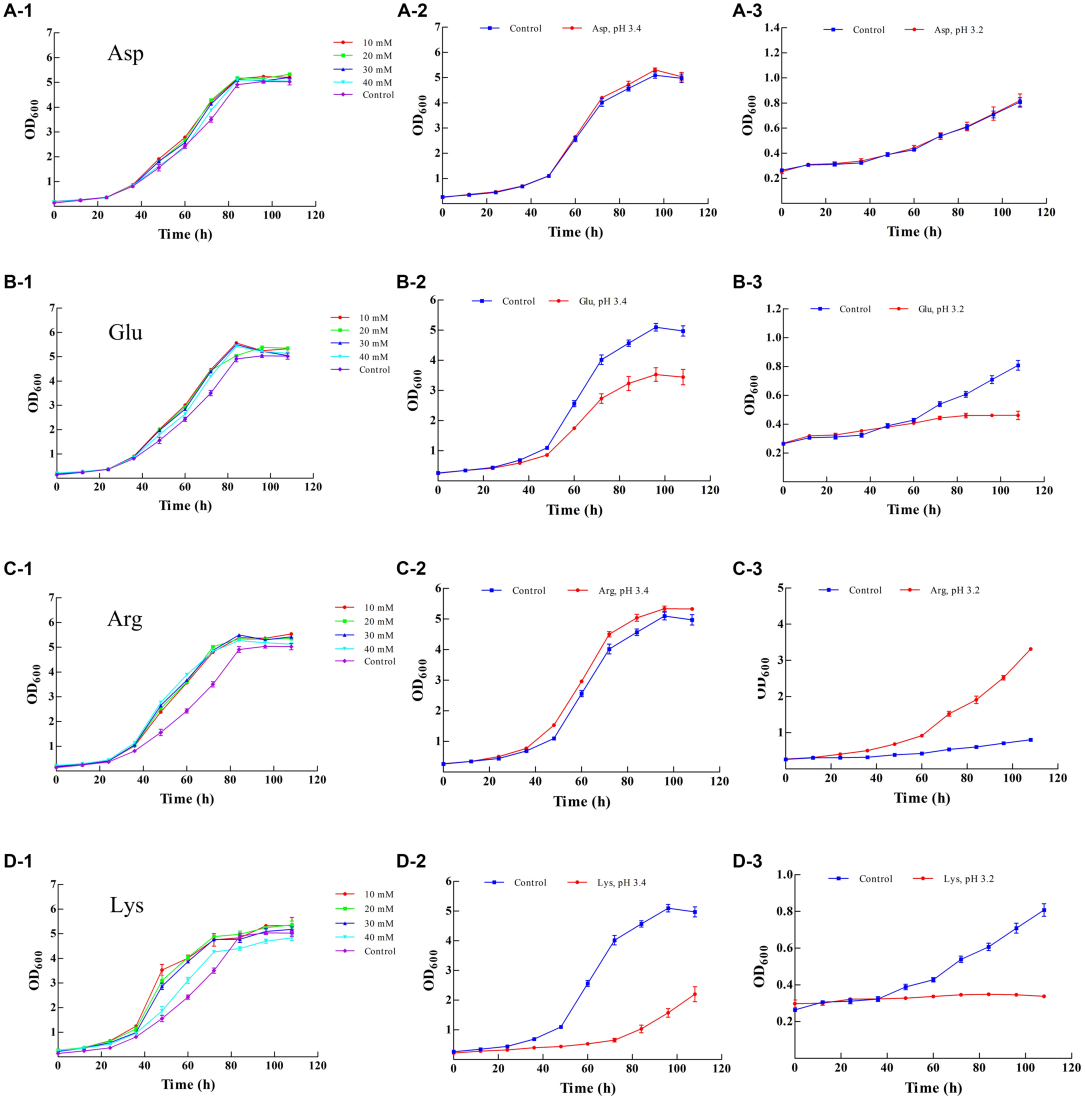
Effect of different amino acids on the growth of Z-1. **(A-1–A-3)**: effect of aspartic acid; **(B-1–B-3)**: effect of glutamic acid; **(C-1–C-3)**: effect of arginine; **(D-1–D-3)**: effect of lysine.

The resistance of LAB to acids can be enhanced by the addition of external substances, such as cysteine, γ-glutamylcysteine, Asp Arg, and sodium L-glutamate, according to previous reports (Huang and Cao, 2012; Jin et al., 2012; Wu et al., 2012a). In the present study, amino acid supplementation was examined. Asp Glu and Lys did not obviously promote the growth of Z-1 at the final cultivation stage (108 h), whereas more biomass was obtained in the presence of Arg compared with the blank control (Figures 2A-1,B-1,C-1,D-1). According to the final biomass, 20 mM Asp 20 mM Glu, 10 mM Arg and 20 mM Lys were selected for further investigation at low pH values.

**Figure 3:**
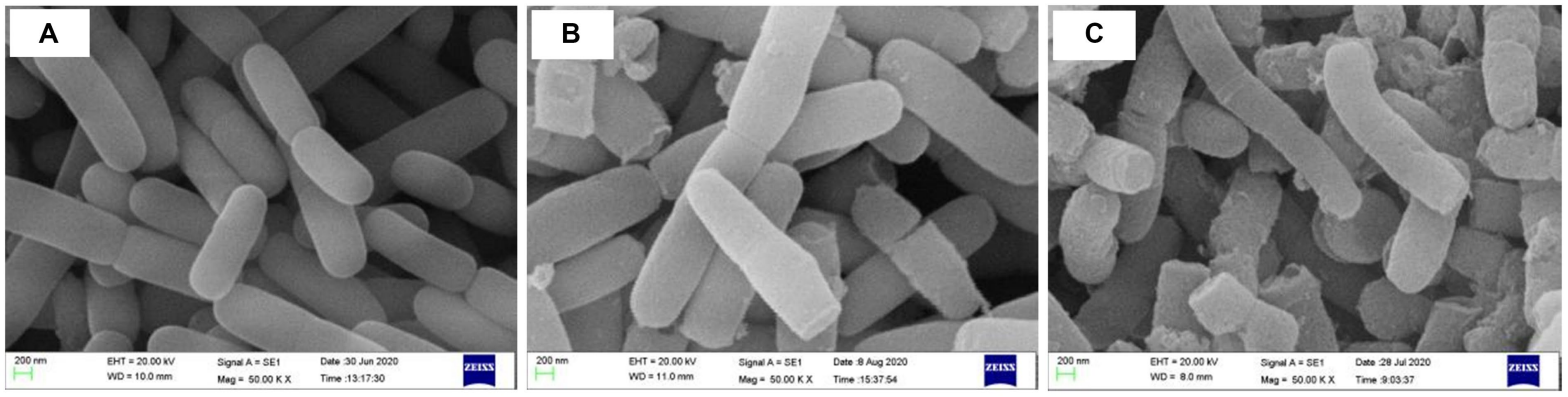
Morphology of Z-1 at different pH values. **(A)**: pH 3.6; **(B)**: pH 3.0; **(C)**: pH 2.5. 5,000×.

As shown in Figures 2A-2,A-3, there was no significant difference in terms of biomass at all tested pH values in the presence or absence of Asp (20 mM), and Z-1 showed increased growth in the absence of Glu and Lys, accompanied by an increase in acidity (Figures 2B-2,B-3,D-2,D-3). In contrast, more biomass was acquired from the culture supplemented with Arg (Figures 2C-2,C-3) than from the control, up to 1.07 times more at pH 3.4 and 4.10 times more at pH 3.2, respectively, suggesting that exogenous Arg can effectively improve Z-1 growth under acid stress. Arg and Asp have been considered to promote *Lactobacillus casei* growth under acid stress (Wu et al., 2012a, 2013). However, Asp did not significantly promote cell growth under acid stress in our work.

### 3.4. Morphological changes in Z-1 under acidic stress

The cellular surface morphology of Z-1 was examined by SEM. As demonstrated in Figure 3, in contrast to the smooth and complete structure at pH 3.6, clearly observable wrinkles and damage appeared after treatment at lower pH values, especially pH 2.5, where most of the bacterial cells were fractured and malformed. Although Z-1 was able to tolerate a certain low-pH environment, it was harmful to the cells to some extent.

### 3.5. Alteration of membrane and intracellular components in Z-1 under acidic stress

**Figure 4:**
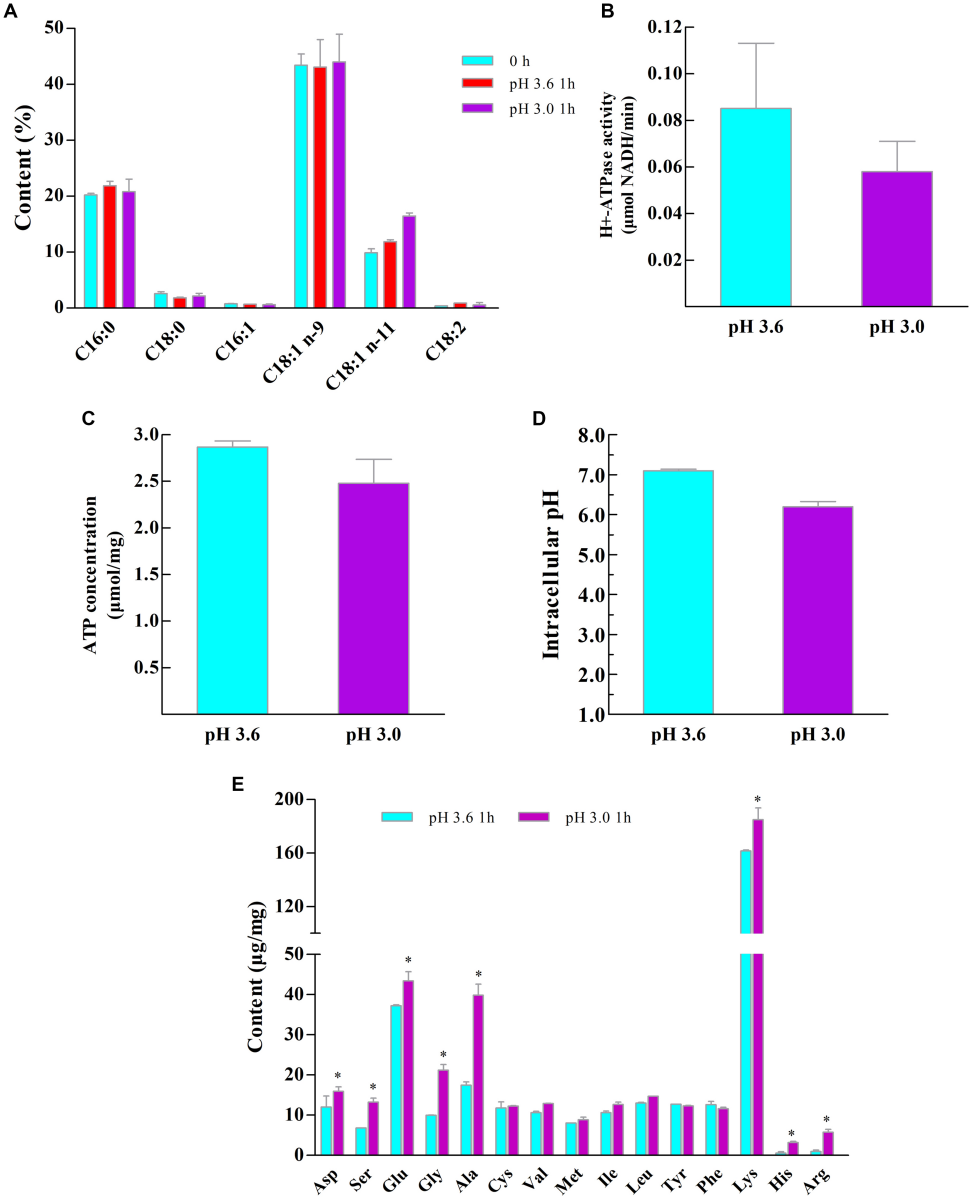
Alterations in the membrane and intracellular components of Z-1 at different pH values. **(A)**: membrane fatty acid content; **(B)**: intracellular H^+^-ATPase concentration; **(C)**: intracellular ATP concentration; **(D)**: intracellular pH; **(E)**: intracellular amino acid composition. ^*^: *p* < 0.05, *n* = 3.

It has been shown that the fluidity of cell membranes can be regulated by altering the ratio of unsaturated fatty acids to saturated fatty acids to resist external environmental stress (Mykytczuk et al., 2007; Rodríguez-Vargas et al., 2007; Kubota et al., 2008; Wu et al., 2012b). As shown in Figure 4A, a total of 6 membrane fatty acids were detected from the Z-1 cell membrane, among which palmitic acid and oleic acid were dominant. In response to acid stress, the contents of C16:0 and C18:0 remained relatively constant at 20 and 2%, respectively, while the relative content of the unsaturated fatty acid C18:1 (n-11) rose from 9.83% (pH 3.6) to 16.46% (pH 3.0) (*p* < 0.05) after 1 h, suggesting that Z-1 adapted to an acidic environment by altering the membrane fatty acid content, which enhanced its cell membrane fluidity (Rodríguez-Vargas et al., 2007; Wu et al., 2012b). In addition, energy production and amino acid metabolism are beneficial for acid tolerance (Koponen et al., 2012; Teixeira et al., 2014; Li et al., 2020). It has been reported that H^+^-ATPase enzymatic activity can reflect the survival rate of LAB under acid stress (Lebeer et al., 2008). As presented in Figures 4B,C, the H^+^-ATPase enzymatic activity was significantly higher (*p* < 0.05) at pH 3.6 than at pH 3.0, and the intracellular ATP concentrations were not much different at these two pH levels. This result may be related to the fact that the optimum pH of H^+^-ATPase activity is slightly acidic (pH 5.0 ~ 5.5), and H^+^-ATPase activity is gradually eliminated in a very low-pH environment (Bender and Marquis, 1987). However, no firm link between Z-1 acid tolerance and H^+^-ATPase activity can be drawn from the results of H^+^-ATPase activity and intracellular ATP concentration.

Maintaining dynamical equilibrium of pH_i_ at the neutral level is an important physiological property of LAB under acid stress. As shown in Figure 4D, pH_i_ is slightly lower at pH 3.0 than at pH 3.6, and cells have a good ability to maintain pH_i_ homeostasis at pH 3.6, with results in agreement with the optimal pH 3.6 for Z-1 growth and a neutral pH for Z-1 non-growth.

Amino acid metabolism plays various physiological roles in LAB, such as regulating intracellular pH, generating ATP or redox force, and resisting external environmental stress (Fernández and Zúñiga, 2006). Apart from the measurements mentioned above, the intracellular amino acids were determined under acid stress (Figure 4E). The intracellular contents of Asp serine (Ser), Glu, glycine (Gly), alanine (Ala), Lys, histidine (His) and Arg were obviously increased compared with those in the control group. In fact, Glu is able to consume H^+^ through decarboxylation (Zhao et al., 2017). The metabolite of Glu, namely, γ-aminobutyric acid (GABA), is able to alleviate acid stress (Higuchi et al., 1997). Nonetheless, Figures 2B-1–B-3 shows that Glu supplementation does not promote bacterial growth; rather, growth is reduced after Glu supplementation, and Glu may not be one of the decisive factors for the acid resistance of Z-1. In addition, a finding is the regulation of Asp and Arg metabolism during acid stress, the metabolic pathway of them may be shifted by increasing the flux from Asp to Arg (arginine deiminase system (ADI)) (Wu et al., 2013). The ADI system has been identified to produce ATP and ammonia to help cells resist acid stress (Teixeira et al., 2014). Meanwhile, Asp is also the precursor of Ala and threonine (Thr), then the Gly is produced through the conversion of Thr, subsequently Ser is produced (Wu et al., 2013).

### 3.6. Transcriptomics analysis related to acid tolerance

**Figure 5:**
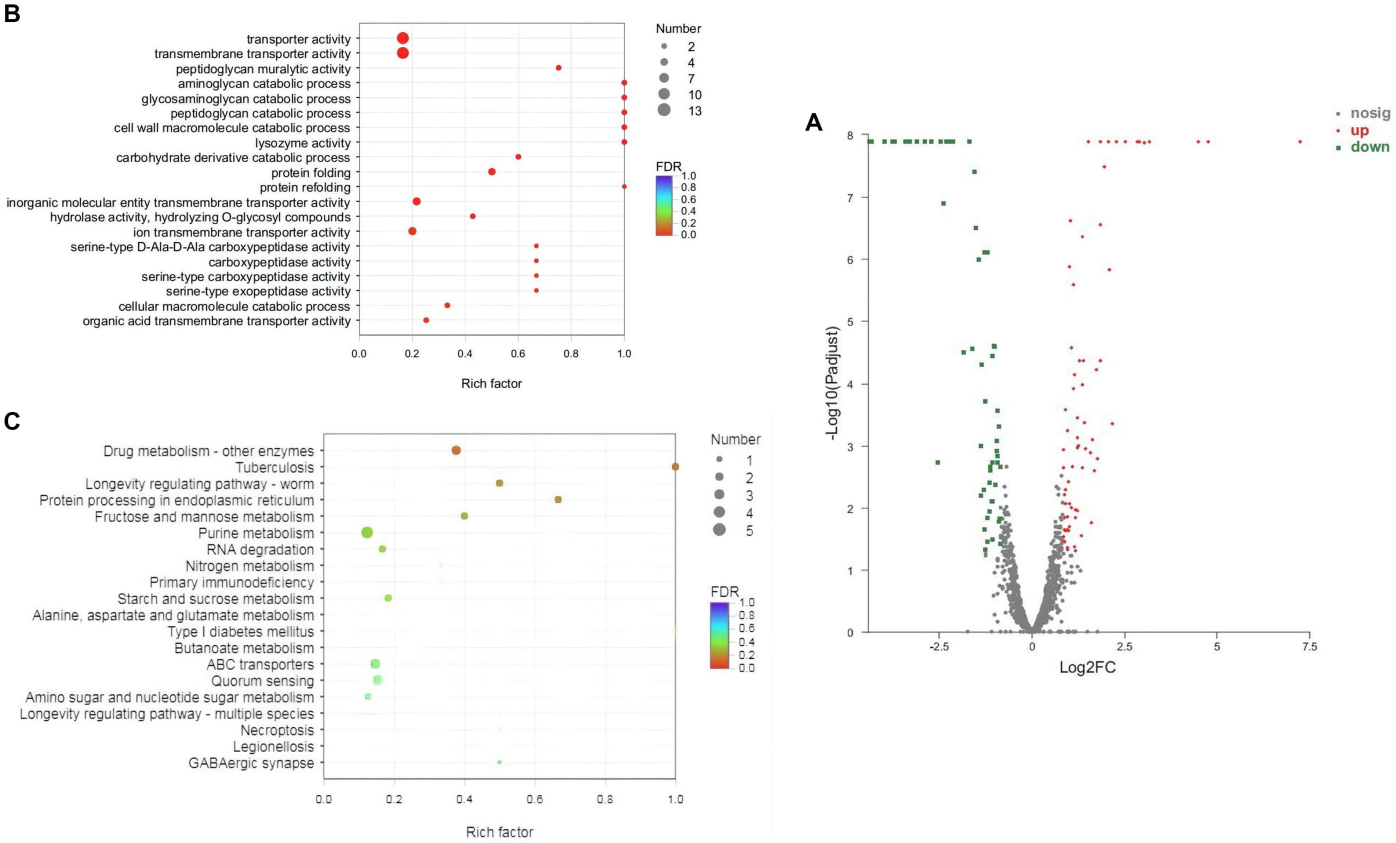
Distribution map and enrichment of differentially expressed genes in Z-1. **(A)**: Distribution map of differentially expressed genes (DEGs); **(B)**: GO enrichment scatter plot analysis; **(C)**: KEGG enrichment scatter plot analysis; the abscissa represents the ratio of the number of DEGs enriched in the pathway to the number of genes annotated in the pathway; the ordinate represents the KEGG entry; the size of the dots indicates the number of DEGs enriched in the pathway; the colour indicates the significant Q value of the pathway.

Transcriptome sequencing was performed to further reveal the acid tolerance characteristics of Z-1. The total number of bases in 6 samples was 2.41 × 10^10^ bp, with a low base error rate. Q20 (%) and Q30 (%) were greater than 97.97 and 93.83%, respectively (Q20 and Q30 represent the percentage of bases with Phred quality score (Q score) greater than 20 and 30 in the total base, respectively), and the rRNA content was far below 15%. Other detailed information is compiled in Supplementary Table S2. The overall transcription levels were quantified by TPM metrics, and 93 genes were differentially expressed in the experimental group compared with the control group, including 51 upregulated genes and 42 downregulated genes (Figure 5A). The transcriptome sequencing data were submitted to the National Center for Biotechnology Information (NCBI) (Accession ID: SRR23071480-SRR23071485).

To further analyse these DEGs, GO and KEGG analyses were conducted (Supplementary Tables S3, S4, Supplementary Figure S1). Accordingly, in terms of enriched GO and KEGG pathways, the DEGs were highly associated with carbohydrate metabolism, amino acid metabolism, ABC transporters, RNA degradation, quorum sensing, and other pathways (Figures 5B,C).

Based on the enrichment analysis described above, acid tolerance was derived from amino acid, energy and lipid metabolism, RNA degradation and nucleotide repair, and signalling pathways.

**Figure 6:**
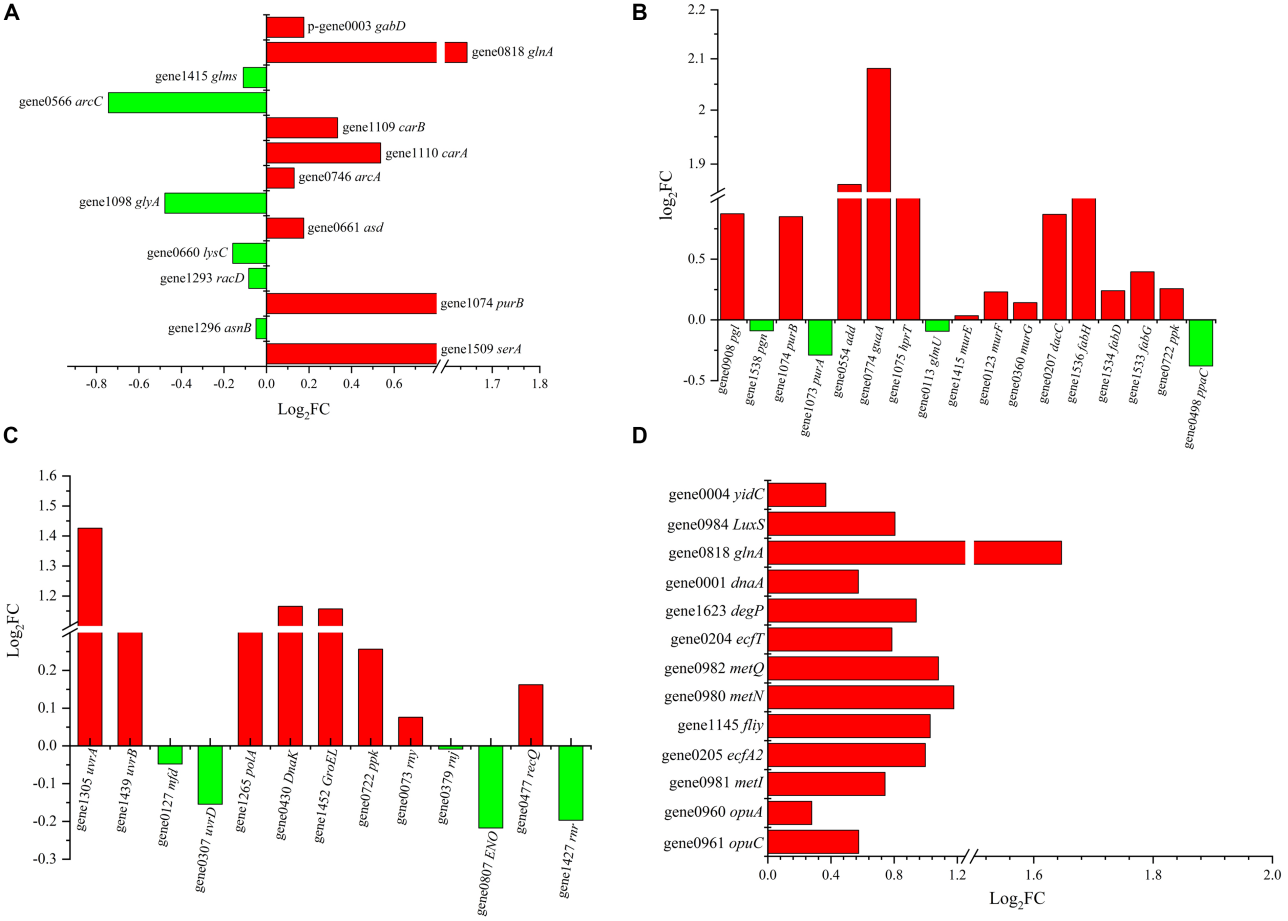
DEGs of different metabolic pathways in Z-1 under acidic stress. **(A)**: amino acid metabolism; **(B)**: energy and lipid metabolism; **(C)**: RNA degradation and nucleotide repair; **(D)**: signalling pathway.

Eight upregulated DEGs enriched in amino acid metabolism were identified, including *gabD*, *glnA*, *carB*, *carA*, *arcA*, *asd*, *purB* and *serA* (Figure 6A). Specifically, *carA*, *carB*, *glnA* and *arcA* are related to the metabolism of Arg, glutamine and citrulline. Glutamine can be converted into Arg and then stepwise converted into citrulline. Then, citrulline is converted to ornithine and carbamoyl phosphate and finally hydrolysed to NH_3_ and ATP. This process not only intracellularly generates the alkaline substance NH_3_ but also provides energy to help resist the external acidic environment. Meanwhile, the *asnB* gene, which is involved in Asp synthesis, was downregulated, which matched well with the aforementioned observation that the addition of Asp did not help Z-1 respond to acid stress.

In fact, energy is indispensable in the response to acidic stress. The results showed that there were 13 upregulated genes related to energy metabolism in Z-1 (Figure 6B). Specifically, *purB*, *guaA* and *hprT* were involved in the nucleotide biosynthesis pathway, and *murE/F/G* was annotated to peptidoglycan synthase, which participates throughout the entire process of peptidoglycan synthesis. Peptidoglycan is an important part of the cell wall in gram-positive bacteria, and its synthesis facilitates cell resistance to pH stress. *fabD*, *fabH* and *fabG* render the activities of 3-oxoacyl-ACP synthase, malonyltransferase and reductase in the lipid metabolism process, which accelerate the metabolism of lipids and constantly provide energy to pump out H^+^. H^+^-ATPase plays an important role in maintaining the stability of intracellular pH (Matsumoto et al., 2004), they can pump H^+^ out through ATP hydrolysis or synthesize ATP by consuming the H^+^ gradient (Nakanishi et al., 2018). However, the gene *ppaC*, which is involved in the transport of protons by H^+^-ATPases, was down-regulated and H^+^-ATPases activity was affected. This result matched well with previous results that H^+^-ATPase activity did not significantly increase, but it is not consistent with existing reports (Fortier et al., 2003; Wu et al., 2013). A reasonable explanation could be that no connection was built between H^+^-ATPase and the acid tolerance of Z-1.

Furthermore, in response to acid stress, some LAB enable protein and nucleic acid repair mechanisms (Cappa et al., 2005; Huang et al., 2011). Transcriptional evidence showed that the *DnaK*, *GroEL*, *rny*, *recQ* and *uvrA* genes were upregulated at different levels (Figure 6C). They are involved in the folding or assembly of proteins, RNA degradation and nucleotide repair, through which Z-1 is available to adapt to acidic stress. *DnaK* and *GroEL* regulate the synthesis of the molecular chaperone proteins DnaK and GroEL, respectively. DnaK can enhance the biosynthesis of F1-F0-ATPase and help remove protons to maintain intracellular pH homeostasis (Kim and Batt, 1993). *rny* and *recQ* are annotated to ribonuclease Y and hypothetical proteins, respectively, which participate in the RNA degradation process and accelerate cell renewal. The *uvrA* gene plays an important role in DNA repair, which is conducive to the adaptation of lactic acid bacteria to acidic environments (Cappa et al., 2005).

Aside from amino acid metabolism and DNA repair, ABC transporters are closely related to acid tolerance (Figure 6D). In this setting, the *opuC* and *opuA* genes, which encode ABC transporter permeases, were upregulated, which possibly helped in the resistance to external stress (Saum and Müller, 2008). In addition, the upregulation of *fliy* and *ecfA2* may help intracellular H^+^ cross the membrane, given the increased activity of ABC transport substrate binding protein and ABC transporter. On the other hand, several reports have suggested that the transmission of stress signals through two-component systems as well as quorum sensing plays vital roles in the bacterial response to environmental stimuli (Mascher et al., 2006). This could explain the fact that the genes *dnaA* and *glnA* were upregulated, which makes cells rapidly proliferate by accelerating DNA duplication under acid stress so that cells can be protected by quorum sensing. *LuxS* is also involved in mediating quorum sensing signals to regulate the acid tolerance of bacteria (Li et al., 2020). Accordingly, the genes *glnA*, *carA/B*, *arcA*, *murE/F/G*, *fabD/H/G*, *DnaK*, *uvrA*, *opuA/C*, *fliy*, *ecfA2*, *dnaA* and *LuxS* were presumed to be the key genes in the acid tolerance mechanism of Z-1.

To validate the reliability and availability of the DEGs obtained from the RNA-Seq analysis, a total of 10 DEGs were randomly selected for qPCR analysis. The results of RT–qPCR analysis were correlated with those of transcriptome analysis (Supplementary Figure S2).

## 4. Conclusion

While LAB play an important role in the formation of flavor in vinegar, several LAB can also cause vinegar deterioration, as their gas-producing properties and acid tolerance exacerbate the difficulties in managing safe vinegar production. Therefore, it is important to grasp the intrinsic factors of acid tolerance in these gas-producing strains. *Acetilactobacillus jinshanensis* subsp. *aerogenes* Z-1 is an acid-tolerant LAB isolated from spoiled Sichuan bran vinegar that can survive for approximately 15 days in 4° and 6° vinegar. The addition of Arg effectively improved the growth performance of Z-1 under acidic conditions, whereas there was no such promotion with Asp., Glu or Lys. Under acidic stress, the relative content of unsaturated fatty acid C18:1 (n-11) was increased, accompanied by intracellular accumulation of Asp., Ser, Glu, Gly, Ala, Lys, His and Arg. Transcriptional evidence showed that amino acid metabolism, energy and lipid metabolism, RNA degradation and nucleotide repair, and signalling pathways were closely related to acid tolerance. Accordingly, the *glnA*, *carA/B*, *arcA*, *murE/F/G*, *fabD/H/G*, *DnaK*, *uvrA*, *opuA/C*, *fliy*, *ecfA2*, *dnaA* and *LuxS* genes were presumed to be the key genes. This work is helpful for the formation of a comprehensive strategy to solve swelling deterioration based on exploration of the acid tolerance mechanism of Z-1.

## Data availability statement

The datasets presented in this study can be found in online repositories. The names of the repository/repositories and accession number(s) can be found at: https://www.ncbi.nlm.nih.gov/, PRJNA922687.

## Author contributions

QL: conceptualization, supervision, formal analysis, writing - original draft. KH: investigation, formal analysis, methodology, writing-review and editing. JM: data curation, investigation, and formal analysis. JL, AL, and YY: formal analysis, methodology, writing-review and editing. XA, LH, SC, LZ, and MG: resources and methodology. SL: conceptualization, supervision, project administration, writing-review and editing, and funding acquisition. All authors contributed to the article and approved the submitted version.

## Funding

This work was supported by the financial support from the Science and Technology Department of Sichuan Province (2023ZHCG0081).

## Conflict of interest

MG is employed by Sichuan Baoning Vinegar Co., Ltd.

The remaining authors declare that the research was conducted in the absence of any commercial or financial relationships that could be construed as a potential conflict of interest.

## Publisher’s note

All claims expressed in this article are solely those of the authors and do not necessarily represent those of their affiliated organizations, or those of the publisher, the editors and the reviewers. Any product that may be evaluated in this article, or claim that may be made by its manufacturer, is not guaranteed or endorsed by the publisher.

## References

[ref1] Al-Dalali S. Zheng F. Sun B. Chen F. (2020). Characterization and comparison of aroma profiles and aroma-active compounds between traditional and modern Sichuan vinegars by molecular sensory science. J. Agric. Food Chem. 68, 5154–5167. doi: 10.1021/acs.jafc.0c00470, PMID: 32281377

[ref2] Bender G. R. Marquis R. E. (1987). Membrane ATPases and acid tolerance of *Actinomyces viscosus* and *Lactobacillus casei*. Appl. Environ. Microbiol. 53, 2124–2128. doi: 10.1128/aem.53.9.2124-2128.1987, PMID: 2445289 PMC204068

[ref3] Breeuwer P. Drocourt J. L. Rombouts F. M. Abee T. (1996). A novel method for continuous determination of the intracellular pH in bacteria with the internally conjugated fluorescent probe 5 (and 6-)-carboxyfluorescein succinimidyl ester. Appl. Environ. Microbiol. 62, 178–183. doi: 10.1128/aem.62.1.178-183.1996, PMID: 16535209 PMC1388751

[ref4] Cappa F. Cattivelli D. Cocconcelli P. S. (2005). The uvrA gene is involved in oxidative and acid stress responses in *Lactobacillus helveticus* CNBL1156. Res. Microbiol. 156, 1039–1047. doi: 10.1016/j.resmic.2005.06.003, PMID: 16125908

[ref5] Fernández M. Zúñiga M. (2006). Amino acid catabolic pathways of lactic acid bacteria. Crit. Rev. Microbiol. 32, 155–183. doi: 10.1080/10408410600880643 16893752

[ref6] Fortier L.-C. Tourdot-Maréchal R. Diviès C. Lee B. H. Guzzo J. (2003). Induction of *Oenococcus oeni* H^+^-ATPase activity and mRNA transcription under acidic conditions. FEMS Microbiol. Lett. 222, 165–169. doi: 10.1016/S0378-1097(03)00299-4, PMID: 12770702

[ref7] Gan X. Tang H. Ye D. Li P. Luo L. Lin W. (2017). Diversity and dynamics stability of bacterial community in traditional solid-state fermentation of Qishan vinegar. Ann. Microbiol. 67, 703–713. doi: 10.1007/s13213-017-1299-6

[ref8] Higuchi T. Hayashi H. Abe K. (1997). Exchange of glutamate and gamma-aminobutyrate in a *lactobacillus* strain. J. Bacteriol. 179, 3362–3364. doi: 10.1128/jb.179.10.3362-3364.1997, PMID: 9150237 PMC179120

[ref9] Huang G. Cao L. Y. (2012). Effect of sodium L-glutamate on growth and survival of *Lactobacillus brevis* NCL912 at different acidic pH. Ann. Microbiol. 62, 351–355. doi: 10.1007/s13213-011-0269-7

[ref10] Huang G. Li C. Cao Y. (2011). Proteomic analysis of differentially expressed proteins in *Lactobacillus brevis* NCL912 under acid stress. FEMS Microbiol. Lett. 318, 177–182. doi: 10.1111/j.1574-6968.2011.02257.x, PMID: 21385203

[ref11] Jin J. Zhang B. Guo H. Cui J. Jiang L. Song S.. (2012). Mechanism analysis of acid tolerance response of *Bifidobacterium longum* subsp. *longum* BBMN 68 by gene expression profile using RNA-sequencing. PLoS One 7:e50777. doi: 10.1371/journal.pone.0050777, PMID: 23236393 PMC3517610

[ref12] Kim S. G. Batt C. A. (1993). Cloning and sequencing of the *Lactococcus lactis* subsp. *lactis* groESL operon. Gene 127, 121–126. doi: 10.1016/0378-1119(93)90626-E, PMID: 8486277

[ref13] Koponen J. Laakso K. Koskenniemi K. Kankainen M. Savijoki K. Nyman T. A.. (2012). Effect of acid stress on protein expression and phosphorylation in *Lactobacillus rhamnosus* GG. J. Proteome 75, 1357–1374. doi: 10.1016/j.jprot.2011.11.009, PMID: 22119544

[ref14] Kubota H. Senda S. Nomura N. Tokuda H. Uchiyama H. (2008). Biofilm formation by lactic acid bacteria and resistance to environmental stress. J. Biosci. Bioeng. 106, 381–386. doi: 10.1263/jbb.106.381 19000615

[ref15] Lebeer S. Vanderleyden J. De Keersmaecker S. C. J. (2008). Genes and molecules of *lactobacilli* supporting probiotic action. Microbiol. Mol. Biol. Rev. 72, 728–764. doi: 10.1128/MMBR.00017-08 19052326 PMC2593565

[ref16] Li S. Li P. Liu X. Luo L. Lin W. (2016). Bacterial dynamics and metabolite changes in solid-state acetic acid fermentation of Shanxi aged vinegar. Appl. Microbiol. Biotechnol. 100, 4395–4411. doi: 10.1007/s00253-016-7284-3, PMID: 26754813

[ref17] Li W. Yang L. Nan W. Lu J. Lv J. (2020). Whole-genome sequencing and genomic-based acid tolerance mechanisms of *Lactobacillus delbrueckii* subsp. *bulgaricus* LJJ. Appl. Microbiol. Biotechnol. 104, 7631–7642. doi: 10.1007/s00253-020-10788-5, PMID: 32715364

[ref18] Mascher T. Helmann J. D. Unden G. (2006). Stimulus perception in bacterial signal-transducing histidine kinases. Microbiol. Mol. Biol. Rev. 70, 910–938. doi: 10.1128/MMBR.00020-06, PMID: 17158704 PMC1698512

[ref19] Matsumoto M. Ohishi H. Benno Y. (2004). H^+^-ATPase activity in *Bifidobacterium* with special reference to acid tolerance. Int. J. Food Microbiol. 93, 109–113. doi: 10.1016/j.ijfoodmicro.2003.10.009, PMID: 15135587

[ref20] Mykytczuk N. C. S. Trevors J. T. Leduc L. G. Ferroni G. D. (2007). Fluorescence polarization in studies of bacterial cytoplasmic membrane fluidity under environmental stress. Prog. Biophys. Mol. Biol. 95, 60–82. doi: 10.1016/j.pbiomolbio.2007.05.001, PMID: 17628643

[ref21] Nakanishi A. Kishikawa J. I. Tamakoshi M. Mitsuoka K. Yokoyama K. (2018). Cryo-EM structure of intact rotary H^+^-ATPase/synthase from *Thermus thermophilus*. Nat. Commun. 9, 140–146. doi: 10.1038/s41467-017-02553-6, PMID: 29311594 PMC5758568

[ref22] Nie Z. Zheng Y. Du H. Xie S. Wang M. (2015). Dynamics and diversity of microbial community succession in traditional fermentation of Shanxi aged vinegar. Food Microbiol. 47, 62–68. doi: 10.1016/j.fm.2014.11.006, PMID: 25583338

[ref23] Rodríguez-Vargas S. Sánchez-García A. Martínez-Rivas J. M. Prieto J. A. Randez-Gil F. (2007). Fluidization of membrane lipids enhances the tolerance of *Saccharomyces cerevisiae* to freezing and salt stress. Appl. Environ. Microbiol. 73, 110–116. doi: 10.1128/AEM.01360-06, PMID: 17071783 PMC1797130

[ref24] Saum S. H. Müller V. (2008). Regulation of osmoadaptation in the moderate halophile *Halobacillus halophilus*: chloride, glutamate and switching osmolyte strategies. Saline Syst. 4:4. doi: 10.1186/1746-1448-4-4, PMID: 18442383 PMC2412884

[ref25] Teixeira J. S. Seeras A. Sanchez-Maldonado A. F. Zhang C. Su M. S.-W. Gänzle M. G. (2014). Glutamine, glutamate, and arginine-based acid resistance in *Lactobacillus reuteri*. Food Microbiol. 42, 172–180. doi: 10.1016/j.fm.2014.03.015, PMID: 24929734

[ref26] Wang C. Cui Y. Qu X. (2018). Mechanisms and improvement of acid resistance in lactic acid bacteria. Arch. Microbiol. 200, 195–201. doi: 10.1007/s00203-017-1446-2 29075866

[ref27] Wang X. Hu K. Liu F. Mou J. Lai J. Zhang M.. (2023). Isolation and characterization of a gas-producing and acid-resistant bacterium from spoiled vinegar. Int. J. Food Microbiol. 394:110167. doi: 10.1016/j.ijfoodmicro.2023.110167, PMID: 36913840

[ref28] Wu C. Zhang J. Chen W. Wang M. Du G. Chen J. (2012a). A combined physiological and proteomic approach to reveal lactic-acid-induced alterations in *Lactobacillus casei* Zhang and its mutant with enhanced lactic acid tolerance. Appl. Microbiol. Biotechnol. 93, 707–722. doi: 10.1007/s00253-011-3757-6, PMID: 22159611

[ref29] Wu C. Zhang J. Du G. Chen J. (2013). Aspartate protects *Lactobacillus casei* against acid stress. Appl. Microbiol. Biotechnol. 97, 4083–4093. doi: 10.1007/s00253-012-4647-2, PMID: 23292549

[ref30] Wu C. Zhang J. Wang M. Du G. Chen J. (2012b). *Lactobacillus casei* combats acid stress by maintaining cell membrane functionality. J. Ind. Microbiol. Biotechnol. 39, 1031–1039. doi: 10.1007/s10295-012-1104-2, PMID: 22366811

[ref31] Yu Y. Li X. Zhang J. Chai L. J. Lu Z. M. Xu Z. H. (2020). *Lactobacillus jinshani* sp. nov., isolated from solid-state vinegar culture of Zhenjiang aromatic vinegar. Antonie Van Leeuwenhoek 113, 43–54. doi: 10.1007/s10482-019-01316-1, PMID: 31407135

[ref32] Zhang J. Du G. C. Zhang Y. Liao X. Y. Wang M. Li Y.. (2010). Glutathione protects *Lactobacillus sanfranciscensis* against freeze-thawing, freeze-drying, and cold treatment. Appl. Environ. Microbiol. 76, 2989–2996. doi: 10.1128/AEM.00026-09, PMID: 20208023 PMC2863433

[ref33] Zhang H. M. Duan G. F. Li J. Y. Wang J. L. Wang R. F. (2016). Isolation and identification of microorganism which led the vinegar gas-producing. *J. Shanxi Agric. Univ.*, *Nat. Sci. Ed.* 36, 595–598. doi: 10.13842/j.cnki.issn1671-8151.2016.08.013, (in Chinese).

[ref34] Zhao X. Wei X. Chen D. Sun L. Feng M. (2017). A review on the mechanism of acid and bile salt resistance of lactic acid bacteria. J. Dairy Sci. Technol. 40, 33–36. doi: 10.15922/j.cnki.jdst.2017.03.008

