# Supplementary material for: Insight into the acid tolerance mechanism of Acetilactobacillus jinshanensis subsp. aerogenes Z-1
Source: Front Microbiol. 2023 Jul 14;14:1226031. doi: 10.3389/fmicb.2023.1226031 (PMC10382275; doi:10.3389/fmicb.2023.1226031)
Supplement: Supplementary file 1 [file Data_Sheet_1.docx]

Frontiers in microbiology

Supplementary material for

**Insight into the acid tolerance mechanism of *Acetilactobacillus jinshanensis* subsp. *aerogenes* Z-1**

Qin Li^a, 1^, Kaidi Hu^a, 1^, Juan Mou^a, 1^, Jianlong Li^a^, Aiping Liu^a^, Xiaolin Ao^a^, Yong Yang^a^, Li He^a^, Shujuan Chen^a^, Likou Zou^b^, Mingye Guo^c^, Shuliang Liu^a,^ *

^a^College of Food Science, Sichuan Agricultural University, Ya’an, Sichuan 625014, People’s Republic of China

^b^College of Resources, Sichuan Agricultural University, Chengdu, Sichuan 611130, People’s Republic of China

^c^Sichuan Baoning Vinegar Co., Ltd, Langzhong, Sichuan 637400, People’s Republic of China

^1^Qin Li, Kaidi Hu and Juan Mou are contributed equally to the work.

^*^Corresponding author at: College of Food Science, Sichuan Agricultural University, Ya’an, Sichuan 625014, People’s Republic of China.

E-mail address: lsliang999@163.com (S. Liu).

**Table S1 RT-qPCR primer**

| Gene | Primer sequence (5′–3′) |
| --- | --- |
| *gene0057* | TAAGAACAATAAGGGCAAGG |
| *gene0057* anti | AGCCGCCGTCATAACTAG |
| *dacC* | GTTGACACTAATGGCTTCC |
| *dacC* anti | CGAGTTTGATGTACCTGCT |
| *danK* | TCCGTTACACTTACAGGC |
| *danK anti* | TGAACAGCAGGAATACGA |
| *clpB* | CGTCGCAGCCGCTAAGATGTC |
| *clpB* anti | TCTGAACACTGATGGTGGCACAAG |
| *patA* | AAAATCGGAAATGCTCGTT |
| *patA* anti | GATGGGTTTAGCGTCGTG |
| *recA* | TCGGTGGAGGTATCAGTC |
| *recA* anti | CGTTTGCCATCATAAGAGTA |
| *guaA* | CGCTAAACATTTCTACGG |
| *guaA* anti | TGAAGTCAGTCATGCTCC |
| *glnA* | GGTGCTAAGTGCCGTCGTG |
| *glnA* anti | CGGAGAAGCGGAAGTCAA |
| *Gene0905* | GGTCTAACGGGATGAAAG |
| *Gene0905* anti | TTAGGACCATCTGGGAAG |
| *uvrA* | GAACCTTACGGAGCCAAACGACAG |
| *uvrA* anti | AGCGACATTAGCAGCGTGTAAGC |
| *16S* | CGGCTACCTTGTTACGACTTCACC |
| *16S* anti | CCCAAAGATAGTGCGGCAACCTC |

**Table S2 RNA-seq data statistics results**

| Sample name | Sequences number | Bases number | Base error rate (%) | Q20(%) | Q30(%) | rRNA content (%) |
| --- | --- | --- | --- | --- | --- | --- |
| D1 | 30240938 | 4342445108 | 0.025 | 98.25 | 94.51 | 0.341 |
| D2 | 24099322 | 3473904664 | 0.025 | 98.1 | 94.11 | 0.328 |
| D3 | 30404436 | 4365655654 | 0.025 | 97.97 | 93.83 | 0.342 |
| S1 | 22574498 | 3172091399 | 0.025 | 98.11 | 94.25 | 0.252 |
| S2 | 28093644 | 4035768220 | 0.025 | 98.06 | 94.04 | 0.252 |
| S3 | 33040824 | 4736177984 | 0.025 | 98.13 | 94.2 | 0.26 |

Note: D represents control group, S represents experimental group, number represents biological repetition; Q20 and Q30 represent the percentage of bases with Phred quality score (Q score) greater than 20 and 30 in the total base, respectively.

**Table S3 Differentially expressed gene information of GO annotation**

| Category | Up | Down |
| --- | --- | --- |
| Biological process | 16 | 13 |
| Cellular component | 17 | 16 |
| Molecular function | 28 | 23 |


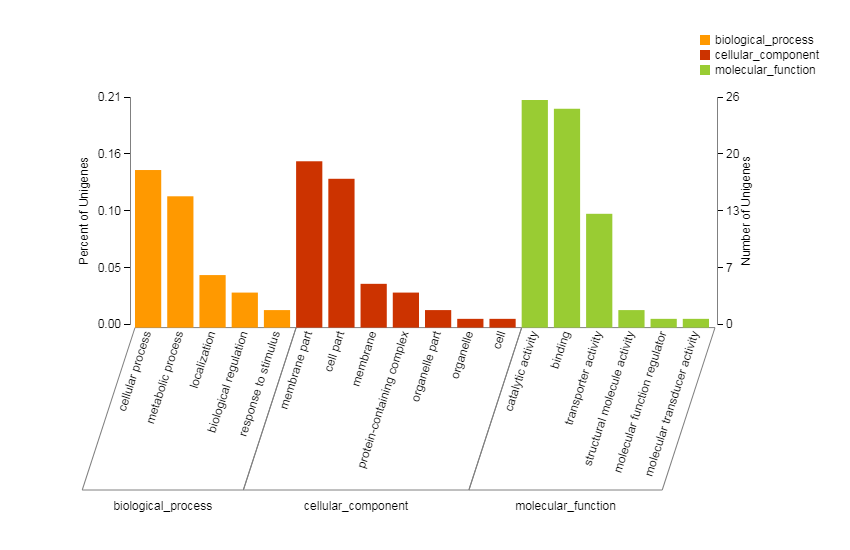


**Figure S1 Differential gene of GO functional annotation**

**Table S4 Differential gene information of KEGG annotation**

| Level | Pathway | Up | Down |
| --- | --- | --- | --- |
| Metabolism | Amino acid metabolism | *gene0861,gene1509,gene1074,gene0501,gene0818* | *－* |
| Metabolism | Carbohydrate metabolism | *gene0818, gene0908* | *gene1017,gene0604,gene1015* |
| Metabolism | Energy metabolism | *gene0818, gene1509* | *－* |
| Metabolism | Glycan biosynthesis and metabolism | *gene0207* | *gene0093* |
| Metabolism | Nucleotide metabolism | *gene0774,gene0773 ,gene0554,gene1075, gene1074* | *－* |
| Metabolism | Lipid metabolism | *gene0796,gene1536* |  |
| Metabolism | Xenobiotics biodegradation and metabolism | *gene0774,gene0773,gene1075* | *－* |
| Genetic Information Processing | Folding, sorting and degradation | *gene0430,gene1452,gene0056,gene0057* | *－* |
| Genetic Information Processing | Replication and repair | *gene1305* | *gene0558* |
| Genetic Information Processing | Translation | *－* | *gene0275,gene0267, gene0480,* |
| Environmental Information Processing | Membrane transport | *gene1145,gene0205,* | *gene0641,gene0639* |
| Environmental Information Processing | Signal transduction | *gene0818* | *－* |
| Cellular Processes | Cell growth and death | *gene0818* | *－* |
| Cellular Processes | Cellular community - prokaryotes | *gene0634* | *gene0904,gene0905,gene0903* |
| Organismal Systems | Aging | *gene0430,gene1452,gene0461* | *－* |
| Organismal Systems | Nervous system | *gene0818* | *－* |
| Human Diseases | Endocrine and metabolic diseases | *gene1452* | *－* |
| Human Diseases | Immune diseases | *gene0554* | *－* |
| Human Diseases | Infectious diseases: Bacterial | *gene0430,gene1452* | *－* |


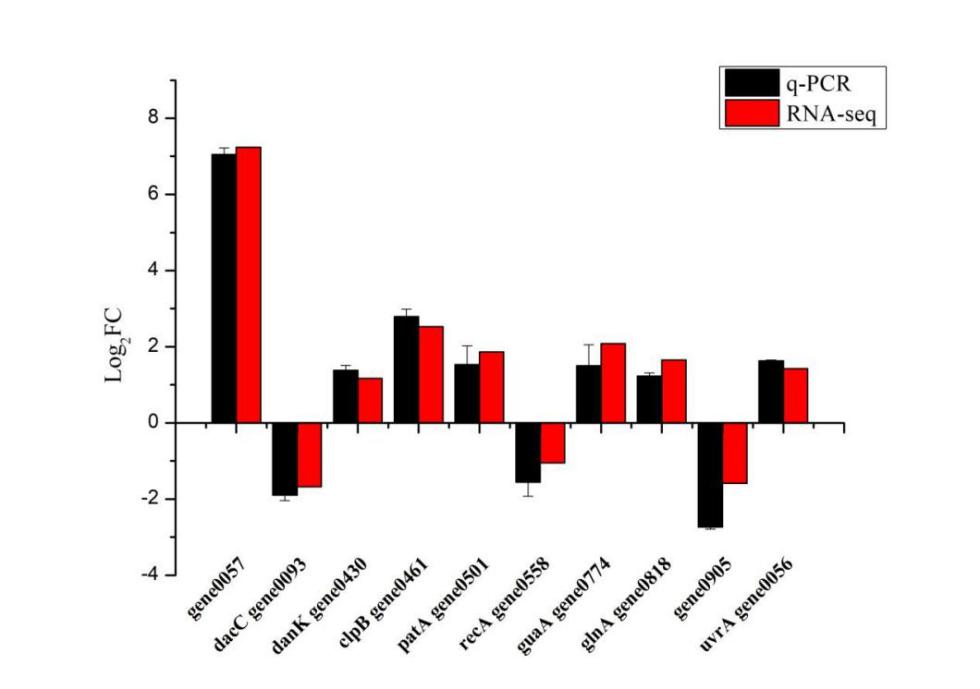


**Figure S2 Result of RT-qPCR**
